# RETRACTION: Evaluating the Efficacy of Recombinant Human Growth Factors in Scar Remodelling for Patients With Facial Soft Tissue Injuries

**DOI:** 10.1111/iwj.70669

**Published:** 2025-04-23

## Abstract

**RETRACTION:** ZhangB. , HeZ. , ZhaoH. , GaoH. , ZhangZ. , GaoZ. , and KeK. , “Evaluating the Efficacy of Recombinant Human Growth Factors in Scar Remodelling for Patients With Facial Soft Tissue Injuries,” International Wound Journal 21, no. 1 (2024): e14649, 10.1111/iwj.14649.38272796 PMC10789918

The above article, published online on 15 January 2024, in Wiley Online Library (http://onlinelibrary.wiley.com/), has been retracted by agreement between the journal Editor in Chief, Professor Keith Harding; and John Wiley & Sons Ltd. Following an investigation by the publisher, all parties have concluded that this article was accepted solely on the basis of a compromised peer review process. The editors have therefore decided to retract the article. The authors did not respond to our notice regarding the retraction.

**RETRACTION:** B. Zhang , Z. He , H. Zhao , H. Gao , Z. Zhang , Z. Gao , and K. Ke , “Evaluating the Efficacy of Recombinant Human Growth Factors in Scar Remodelling for Patients With Facial Soft Tissue Injuries,” International Wound Journal 21, no. 1 (2024): e14649, 10.1111/iwj.14649.38272796 PMC10789918

The above article, published online on 15 January 2024, in Wiley Online Library (http://onlinelibrary.wiley.com/), has been retracted by agreement between the journal Editor in Chief, Professor Keith Harding; and John Wiley & Sons Ltd. Following an investigation by the publisher, all parties have concluded that this article was accepted solely on the basis of a compromised peer review process. The editors have therefore decided to retract the article. The authors did not respond to our notice regarding the retraction.

